# Differences of Microglia in the Brain and the Spinal Cord

**DOI:** 10.3389/fncel.2019.00504

**Published:** 2019-11-14

**Authors:** Fang-Ling Xuan, Keerthana Chithanathan, Kersti Lilleväli, Xiaodong Yuan, Li Tian

**Affiliations:** ^1^Department of Physiology, Faculty of Medicine, Institute of Biomedicine and Translational Medicine, University of Tartu, Tartu, Estonia; ^2^Department of Neurology, Kailuan General Hospital, North China University of Science and Technology, Tangshan, China; ^3^Psychiatry Research Centre, Beijing HuiLongGuan Hospital, Peking University, Beijing, China

**Keywords:** microglia, spinal cord, brain, neuroinflammation, neurological diseases

## Abstract

Microglia were previously regarded as a homogenous myeloid cell lineage in the mammalian central nervous system (CNS). However, accumulating evidences show that microglia in the brain and SC are quite different in development, cellular phenotypes and biological functions. Although this is a very interesting phenomenon, the underlying mechanisms and its significance for neurological diseases in association with behavioral and cognitive changes are still unclear. How microglia differ between these two regions and whether such diversity may contribute to CNS development and functions as well as neurological diseases will be discussed in this Perspective.

## Introduction

Microglia are important for building and defending the central nervous system (CNS) (Tay et al., 2017) are considered the most dynamic glial cell type in the CNS to sense and adapt to their surroundings, thereby giving them a complex feature of heterogeneity (Nayak et al., 2014). However, the implication of spatial heterogeneity of microglia in normal CNS functions and diseases is still evasive. Although the past years have witnessed a dramatic growth of attention on microglial functions in the CNS, evidence available for this Perspective is rather limited, because most microglial studies have investigated only the brain and have used disease models concerning the brain or the spinal microglial functions separately, and a direct comparison between the brain and the spinal cord (SC) under basal or diseased condition is rare. The aim of this paper is hence to provide an updated available knowledge on microglial differences between the brain and the SC and to discuss its potential application for treating neurological diseases. Besides region-specific features, microglia are also increasingly known to differ temporally and between genders, but these other heterogenous aspects will not be focused here and readers are referred to other recent reviews (Mapplebeck et al., 2016; Silvin and Ginhoux, 2018; Rahimian et al., 2019).

## Differences in Development and Phenotypes of Microglia in the Brain Versus in the SC

Using various immunohistochemical markers for microglia, including tomato lectin, CD45, CD68, major histocompatibility complex (MHC)-II, CD11b/c (OX-42), and ionized calcium-binding adaptor molecule-1 (Iba1), researchers have observed generally similar developmental aspects of microglia in their entrance and colonization in the brain and the SC, but minor differences exist (Rezaie and Male, 1999). In a study comparing cell populations in the lumbar locomotor region of the spinal cord and in the brainstem motor nucleus of new-born rats, Cifra et al. (2012) observed less than 10% of microglia in the neonate brainstem but very few microglia in the spinal gray matter (Table 1). Moreover, an earlier study discovered that microglial density in the adult colony-stimulating factor (CSF)1-deficient mice was specifically decreased by 86.4% in the white matter tract of the spinal dorsal column compared with that in age-matched wild-type controls, whereas the decrease was much less (24.0%) in the gray matter of cerebral cortex (Kondo and Duncan, 2009). This suggests that spinal microglia respond to CSF1-dependent cell proliferation more sensitively than cortical microglia, but whether this is due to the differential expression of CSF1 and/or its receptor in different CNS regions is unclear. A very recent study comparing cerebellar and cerebral microglia in CSF1-deficient mice also confirmed that CSF1 depletion did not affect microglia in the forebrain (Kana et al., 2019). Nevertheless, CSF1 is more enriched in the brain than the spinal cord. IL-34, an alternative ligand for CSF1R, is also highly expressed in the brain compared to the spinal cord and is critical for developing cerebral microglia, in contrast to CSF1 (Greter et al., 2012; Wang et al., 2012). However, whether it is dispensable for spinal microglia development or not is unclear. In another recent study using a diphtheria toxin receptor (DTR)-based genetic depletion approach for adult mice, Bruttger et al., 2015) further revealed that while microglia were rapidly ablated in all studied areas, e.g., the cortex, cerebellum and SC, within 3 days, residual microglia recovered more rapidly in the SC than in the cortex within 7 days, providing another evidence on the sensitivity of spinal microglia to changes in environmental cues.

**TABLE 1:** Differences in regional features of brain versus spinal microglia.

**Features**	**Brain versus SC**	**References**
Entrance	• <10% of total brainstem cells but few microglia in the SC in rats at P4; • Entrance in the cerebrum at GW4-8 but in the SC at GW9-16 in humans;	Monier et al., 2007; Cifra et al., 2012
Proliferation	• Spinal microglia more sensitive to CSF1 and genetic ablation; • Cerebral microglia less responsive to CSF1;	Kondo and Duncan, 2009; Bruttger et al., 2015; Kana et al., 2019
Receptors and genes	• Lower expression of CSF1 and IL34 in the SC; • Higher levels of CD11b, CD45, CD86, CCR9 and MHCII but lower level of CD172a in the SC; • *Fos* and *Kif* genes more enriched in the SC; • CST3^–^SPARC^–^IBA1^+^ microglia more enriched in the juvenile SC;	de Haas et al., 2008; Olson, 2010; Wang et al., 2012; Li et al., 2016; Gosselin et al., 2017; Masuda et al., 2019
Response to injury	• *CD68*, *C1qb*, *C3*, *C4a* and *Tgfb1* upregulated in the SC but downregulated in the cortex after SNI; • BDNF upregulated in the SC but downregulated in the hippocampus and no difference in change of TNF after SNI; • Inflammatory response greater in the SC after trauma.	Schnell et al., 1999; Batchelor et al., 2008; Li et al., 2016; Liu et al., 2017

Importantly, much of the knowledge on developmental features of microglia in the SC comes from previous studies on human post-mortem samples (Table 1). In the developing human CNS, both brain and SC microglial progenitors are suggested to enter their respective eminence from the meninges (Rezaie and Male, 1999; Monier et al., 2007). However, Rezaie and Male (1999) found that human amoeboid-like microglia-macrophages appeared in the SC at gestational weeks (GW)9 and peaked at GW16, which is later than entrance in the cerebrum at GW4-8 as described by Monier et al. (2007). Morphologically, Hutchins et al. found that during GW18-24, gray matter microglia were ramified while white matter microglia were amoeboid in the SC (Hutchins et al., 1992). Since morphological maturation of microglia- macrophage precursors stages a step-wise amoeboid, intermediate, and ramified transition (Verney et al., 2010), this indicates an inside-out migratory route of microglia-macrophage progenitors in the SC, which also resembles the early developmental cerebrum (Monier et al., 2007; Verney et al., 2010). McKay et al. found that morphologies of microglia even differed between the dorsal and ventral horns within the spinal gray matter in adult rats (McKay et al., 2007), with both microglial size (length between the tips of the two longest processes through the soma) and soma area smaller in the dorsal horn (DH) than in the ventral horn.

Other microglial phenotypes may also differ from those in the brain at adulthood (Table 1). Fundamental gene expression differences in microglia between the brain and SC have been particularly noticed. For instance, de Haas et al. made a pilot comparison of the protein expression of several microglial molecules among the mouse hippocampus, cerebral cortex, cerebellum and SC, and observed that levels of CD11b, CD45, CD86, and CCR9 were higher in the SC as compared to other regions (de Haas et al., 2008). A later study similarly found higher levels of several immune molecules on spinal microglia than their counterparts in the brain both at steady state and upon viral infection (Olson, 2010). We previously also confirmed that microglial expression of CD11b/c and MHCII were constitutively higher in the SC than in the cerebral cortex and thalamus, which conversely expressed more abundantly CD172a (SIRPα), an inhibitory immune receptor (Li et al., 2016).

Using genome-wide chromatin gene expression profiling and/or single-cell RNA sequencing (scRNAseq) analysis, three recent pivotal studies included microglia in the SC (Matcovitch-Natan et al., 2016; Gosselin et al., 2017). Matcovitch-Natan et al. (2016) provided seminal evidence that microglia underwent a three-step developmental program - early, pre-, and adult microglia across the mouse brain. Gosselin et al. (2017) examined the transcriptomes and epigenetic landscapes of human microglia isolated from surgically resected brain tissue *ex vivo* and after transition to an *in vitro* environment, and studied transcriptomes of various mouse CNS regions. However, neither study specifically addressed regional aspects of spinal microglial transcriptomic features in development and adulthood. In the study of Gosselin et al., gene family members of *Fos* and *Kif* seemed to be more enriched in the SC than the brain as a function of development in mice (Gosselin et al., 2017). Very recently, Masuda et al. (2019) made a thorough scRNAseq analysis of mouse microglia across different CNS regions, including the SC, in embryonic, juvenile and adult stages and found that compared to juvenile microglia, adult microglial clusters showed a more homogenous distribution across regions. Although the authors did not focus on the SC in particular either, their results demonstrated that a minor cluster (C7, representing CST3^–^SPARC^–^IBA1^+^ microglia) was more prevalent in the juvenile SC and cerebellum as well as in the adult cerebellum and corpus callosum compared to the cortex and hippocampus (Masuda et al., 2019). These studies nevertheless provide some first clues on whether differentiation of microglia is orchestrated synchronically in different CNS regions across different developmental stages.

**FIGURE 1:**
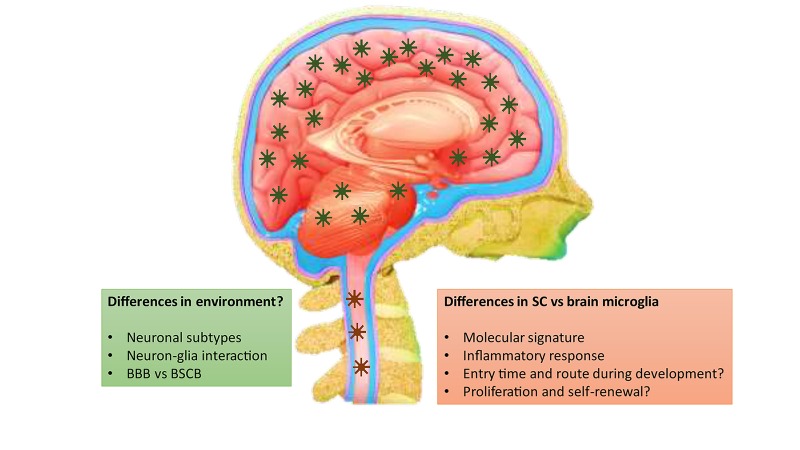
Differences between brain and spinal microglia and the potential contributing factors.

It is still unclear what underlies the fundamental difference between spinal and brain microglia and whether this is mainly caused by intrinsic or extrinsic factors (Figure 1). Guiding cues that drive the differences of microglia between the brain and the SC may come from the peripheral circulation, as blood-derived molecules penetrate the blood-brain barrier (BBB) and the blood-SC barrier (BSCB) in different manners (Abbott et al., 2010; Bartanusz et al., 2011). The BSCB is generally considered as the morphological extension of the BBB into the SC. Nevertheless, evidences suggest that structural and functional differences exist between them, so that the BSCB has higher permeability and lower expressions of tight junction proteins, such as ZO-1 and Occludin, and adherence junction proteins, such as VE-cadherin and β-catenin (reviewed in Bartanusz et al., 2011). It is noteworthy that glial cell types themselves may contribute to the morphological and functional differences between the BBB and BSCB.

## Microglial Regional Heterogeneity in Neuroinflammatory CNS Diseases

Although unconfirmed yet, it is highly expected that basal differences of microglia between the SC and the brain may contribute to the onset, development and treatment response of respective CNS diseases. Such differences are parallel to the facts that the brain and the SC play non-redundant roles in high-order functions of vertebrates and hence require different neurotransmission signals and regional connections. Knowledge on spinal microglia-mediated neuroinflammation and in comparison to brain microglia, however, has been mostly derived from studies on neurological conditions so far, namely traumatic brain injury (TBI), spinal cord injury (SCI), as well as spinal nerve injury models (Details on the respective models are provided in these recent reviews (Zhang and Gensel, 2014; Gensel and Zhang, 2015; Hu et al., 2015; Martini and Willison, 2016; Jassam et al., 2017; Cox, 2018; Table 1). Subtle to dramatic pathological and neuroinflammatory differences exist among different models. For instance, TBI is regarded as a diffuse injury, whereas spinal cord injury and stroke are anatomically discrete (Cox, 2018). Whether microglia contribute to such differences is currently unknown. In a recent study using spared nerve injury (SNI) model in mice, Liu et al. showed opposite changes in dendritic lengths and spine densities between hippocampal CA1 pyramidal neurons and spinal neurons along with differential expression of hippocampal and spinal brain-derived neurotrophic factor (BDNF) after SNI, which was prevented by genetic deletion of tumor necrosis factor (TNF) receptor 1, a microglia-enriched cytokine receptor, and by inhibition or ablation of microglia (Liu et al., 2017). It is intriguing how the same TNF receptor can play opposite roles in regulation of neurons at different sites but differential microglial reaction after SNI would be expected. In line with this notion, using a similar SNI rat model, we earlier had observed that baseline differences of spinal and cortical microglia in CD172a^+^ and MHCII^+^ subpopulations as well as in expression of microglial activation genes underlined their differential responses to SNI as well as to the analgesic effect of minocycline, so that genes involved in M2 polarization and phagocytosis were upregulated in the spinal dorsal horn after SNI compared to Sham, but were downregulated in the cortex (Li et al., 2016). However, Liu et al. found that SNI increased TNF-α levels in both the hippocampus and the ipsilateral spinal dorsal horn compared with shams, which was also similarly dampened by minocycline treatment and by DTR-mediated genetic depletion of microglia (Liu et al., 2017), implying similar activation responses of spinal and hippocampal microglia to SNI. Causes for discrepancies in these studies need to be further examined.

Studies on TBI and SCI have reported that acute inflammatory response to traumatic injury is significantly greater in the SC than in the cerebral cortex. A more careful dissection on these differences was thoroughly provided in an earlier review (Zhang and Gensel, 2014) and therefore we will not reiterate here but only give a couple of exemplar evidences briefly. For instance, Schnell et al. originally found that myeloid recruitment to the lesion site and the surrounding parenchyma was significantly higher in the SC than in the brain. The area of BBB breakdown was substantially larger and vascular damage persisted longer in the SC (Schnell et al., 1999). A later study consistently found that, one week after mechanical injuries to both the gray and white matters, microglial response was significantly greater in the SC compared to the brain (Batchelor et al., 2008). In addition, a greater inflammatory response in the white matter compared to the gray matter within both the brain and SC was observed (McKay et al., 2007; Batchelor et al., 2008). In an *in vitro* cell culture study on morphological properties and secretion of inflammatory and trophic effectors by microglia derived from the brain or spinal cord of neonatal rats, Baskar Jesudasan et al. (2014) demonstrated that spinal microglia assumed a less inflammatory phenotype after lipopolysaccharide-induced activation, with reduced release of proinflammatory cytokines and nitric oxide, a less amoeboid morphology, and reduced phagocytosis relative to brain-derived microglia. These results suggest that local instead of global microglia-modulatory and/or anti-inflammatory strategies targeting microglia more specifically may be more valuable to reduce damages caused by local microglial activation in treating SC-related neurological diseases.

## Discussion

Brain microglia may be more important for regulating neuron-mediated cognition and emotion, whereas spinal microglia more relevant for controlling neuron-mediated sensory-motor functions. This may commit brain and spinal microglia to different CNS conditions, e.g., psychiatric and mental disorders versus sensory-motor neurological diseases. It is doubtless that more and more researchers are recognizing the existence of microglial regional specificity, but more vigorous investigations are still required for us to more deeply understand this interesting phenomenon.

## Author Contributions

All authors listed have made a substantial, direct and intellectual contribution to the work, and approved it for publication.

## Conflict of Interest

The authors declare that the research was conducted in the absence of any commercial or financial relationships that could be construed as a potential conflict of interest.
